# Synthesis of Silver Nanoparticles and Gold Nanoparticles Used as Biosensors for the Detection of Human Serum Albumin-Diagnosed Kidney Disease

**DOI:** 10.3390/ph17111421

**Published:** 2024-10-24

**Authors:** Tiarpa Thongwattana, Ronnakorn Chaiyo, Khanittha Ponsanti, Benchamaporn Tangnorawich, Patcharee Pratumpong, Surachet Toommee, Ratchapol Jenjob, Su-Geun Yang, Yardnapar Parcharoen, Sitakan Natphopsuk, Chiravoot Pechyen

**Affiliations:** 1 Department of Materials and Textile Technology, Faculty of Science and Technology, Thammasat University, Pathum Thani 12120, Thailand; 2 Department of Physics, Faculty of Science and Technology, Thammasat University, Pathum Thani 12120, Thailand; 3 Thammasat University Center of Excellence in Modern Technology and Advanced Manufacturing for Medical Innovation, Thammasat University, Pathum Thani 12120, Thailand; 4 Industrial Arts Program, Faculty of Industrial Technology, Kamphaeng Phet Rajabhat University, Kamphaeng Phet 62000, Thailand; 5 BK21 FOUR Program in Biomedical Science and Engineering, Department of Biomedical Science, Inha University College of Medicine, Incheon 22212, Republic of Korea; 6 Inha Institute of Aerospace Medicine, Inha University College of Medicine, Incheon 22332, Republic of Korea; 7 Chulabhorn International College of Medicine, Thammasat University, Pathum Thani 12120, Thailand

**Keywords:** silver nanoparticles, gold nanoparticles, green synthesis, Phulae pineapple, human serum albumin (HSA), carbon screen-printed electrode

## Abstract

**Background/Objectives:** This study aims to develop a screen-printed carbon electrode (SPCE) modified with silver nanoparticles (AgNPs) and gold nanoparticles (AuNPs) for the detection of human serum albumin (HSA). The objectives include utilizing green synthesis methods for nanoparticle production and evaluating the electrochemical performance of the modified electrodes. **Methods:** AgNPs and AuNPs were synthesized using Phulae pineapple peel extract (PPA) as a reducing agent. The nanoparticles were characterized using UV-visible spectrophotometry (UV-vis), Fourier transform infrared spectroscopy (FTIR), X-ray diffraction (XRD), and transmission electron microscopy (TEM). The electrochemical performance of AgNP/SPCE and AuNP/SPCE was assessed by cyclic voltammetry (CV) studies, and the electrodes were functionalized with anti-HSA antibodies for HSA detection. **Results:** Characterization revealed spherical nanoparticles ranging from 10 to 30 nm. Both AgNP/SPCE and AuNP/SPCE demonstrated improved electrochemical performance compared to bare SPCEs. The modified sensors could detect serum albumin concentrations from 10 to 400 μg/mL, with high correlation values of 0.97 and 0.99 for AgNPs and AuNPs, respectively. **Conclusions:** This research demonstrates the potential of using agricultural waste for green synthesis of nanoparticles and highlights the application of AgNPs and AuNPs in developing sensitive biosensing platforms for the detection of human serum albumin.

## 1. Introduction

Human serum albumin (HSA) is a critical protein found in blood plasma, serving as an important biomarker for kidney health. When HSA leaks into urine due to kidney damage, it indicates the presence of microalbumin, a significant early warning sign of kidney disease. This condition is characterized by urinary albumin excretion levels from 30–300 mg/day from a 24 h urine collection or 30–300 mg/L from a random or first-morning urine sample [1,2,3]. Traditional analytical methods for measuring microalbumin, including fluorescence immunoassays [4,5], radioimmunoassays [6], high-performance liquid chromatography (HPLC) [7], and others [8,9,10,11,12,13], often present challenges such as high costs, prolonged analysis times, low sensitivity, and complex pre-treatment procedures [14].

Electrochemical biosensors have emerged as a promising alternative due to their high sensitivity, stability, low detection limits, cost-effectiveness, and ease of use [15,16]. As a result, there has been a growing interest in the development of electrochemical biosensors for albumin measurement [17,18,19,20,21,22,23,24,25]. Metal nanoparticles, particularly silver (Ag) and gold (Au), are widely used in constructing electrochemical sensors due to their excellent electrical conductivity, catalytic properties, and biocompatibility [26,27]. While these nanoparticles can be synthesized using physical or chemical methods, such approaches often involve high costs, significant energy consumption, and the use of toxic solvents, posing environmental and health risks [28]. In contrast, biological or green synthesis methods are environmentally friendly, cost-effective, and safe, utilizing plant extracts as reducing agents [29,30]. Previous studies have demonstrated the potential of various plant components, including fruits, seeds, and peels, in the synthesis of AgNPs [31,32] and AuNPs [33,34,35].

Thailand, being a tropical country, produces a diverse range of fruits, with pineapple being one of its major agricultural products. The ‘Phulae’ variety of pineapple, known for its unique taste and crisp texture, is highly sought after both domestically and internationally [36,37]. Despite being considered agricultural waste, pineapple peels are rich in bioactive compounds that can facilitate the reduction of metal ions and stabilize the resulting nanoparticles [38,39,40,41].

This study aims to synthesize AgNPs and AuNPs using an eco-friendly biological method that not only addresses the issue of agricultural waste but also enhances the value of such waste products. Specifically, we employ Phulae pineapple peel extract as both the reducing and stabilizing agent in the synthesis of these nanoparticles. The synthesized nanoparticles are subsequently used to modify electrodes designed for electrochemical biosensing applications. We investigate the electrochemical response of the modified electrodes through cyclic voltammetry (CV) and assess their performance as highly sensitive biosensors for the detection of HSA using square-wave voltammetry (SWV).

## 2. Results and Discussion

### 2.1. Characterization of AgNPs and AuNPs Synthesized Using PPA

**Figure 1:**
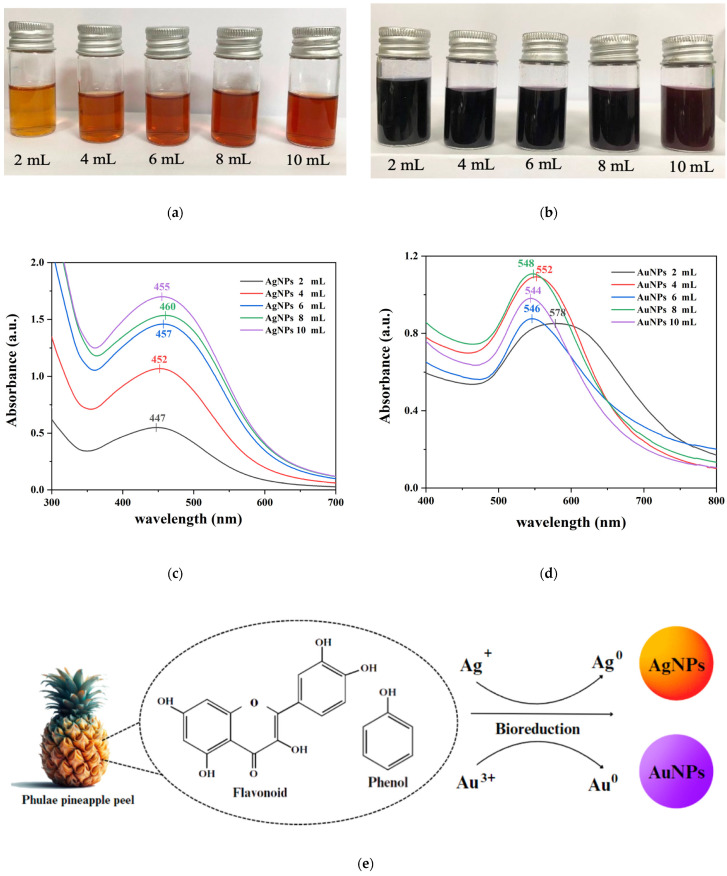
Photographs show the color changes in the solution of (**a**) AgNPs and (**b**) AuNPs formed at different PPA quantities. UV-vis absorption spectra of biosynthesized (**c**) AgNPs and (**d**) AuNPs at different PPA quantities. (**e**) Mechanism of AgNP and AuNP formation using PPA.

The initial indicator showing that PPA was an effective agent for the synthesis of AgNPs and AuNPs was the visual color change that occurred after PPA was added to the solution. The color of the AgNP solution changed to orange-brown (Figure 1a), indicating that AgNPs were formed by the reduction of silver ions (Ag^+^) into silver particles (Ag^0^). The samples show an optical absorption band peak ranging from 440–460 nm (Figure 1c) [42,43,44]. Similarly, the color of the AuNP solution changed to purple (Figure 1b), indicating that AuNPs were formed by the reduction of gold ions (Au^3+^) into gold particles (Au^0^). The samples show an optical absorption band peak ranging from 540–580 nm (Figure 1d) [45,46,47]. These typical characteristic absorption bands for metallic Ag and Au nanoclusters were due to surface plasmon resonance (SPR).

**Figure 2:**
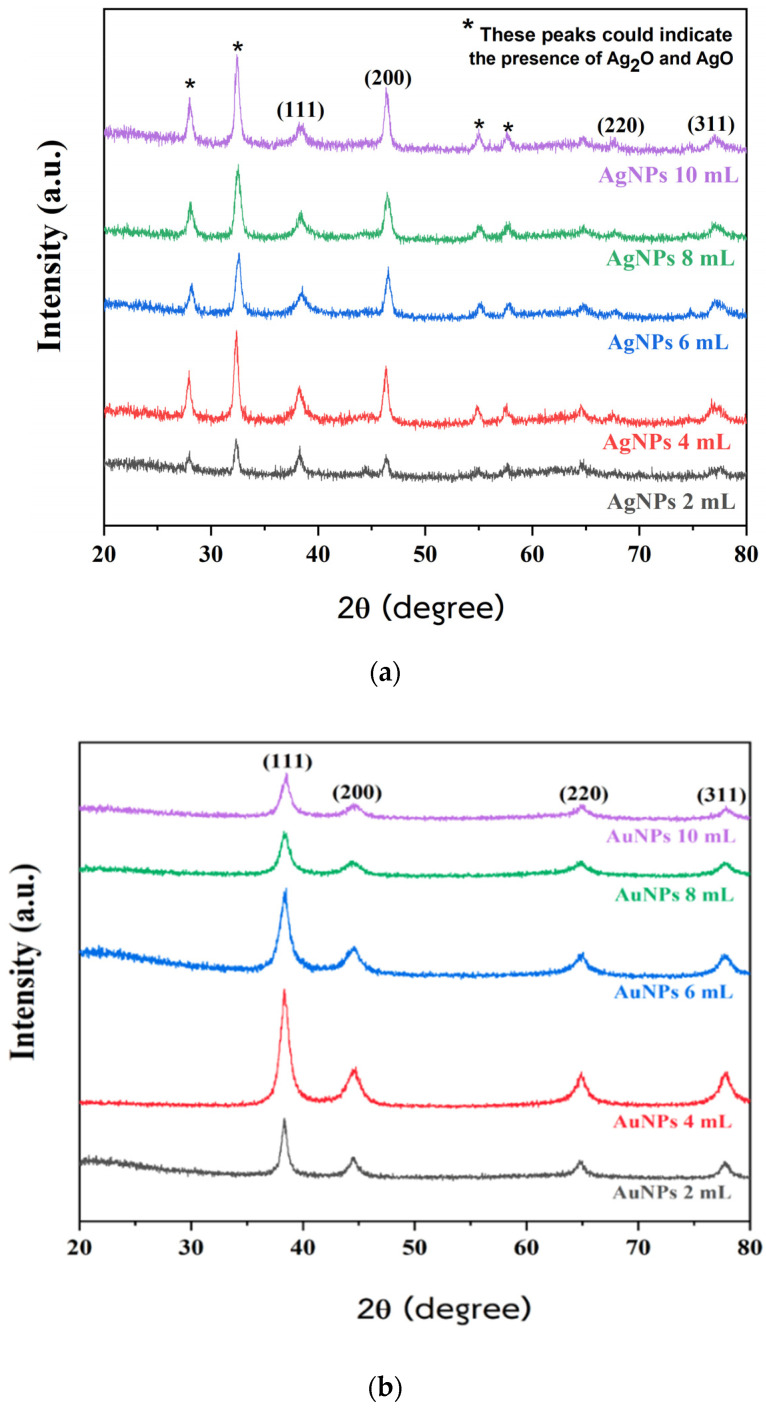
XRD pattern of biosynthesized (**a**) AgNPs and (**b**) AuNPs at different PPA quantities.

The XRD patterns of the crystalline structures of biosynthesized AgNPs and AuNPs using PPA at different PPA quantities are shown in Figure 2a,b. Both have XRD peaks at 2θ degrees that are evident approximately at 38°, 44°, 64°, and 77°, which correspond to the (111), (200), (220), and (311) planes of a face-centered cubic (FCC) structure. These peaks agree with standard report values from the Joint Committee on Powder Diffraction Standards (JCPDS): JCPDS No. 04-0783 for Ag [48,49,50], and JCPDS No. 04-0784 for Au [51,52]. However, some other peaks observed in the diffraction pattern, marked by asterisks* in Figure 2a, were detected at 2θ degrees of 28°, 32°, 54°, and 57°. These peaks could indicate the presence of Ag_2_O and AgO. It is assumed that these peaks are related to residual reducing agents” or might be due to phytochemicals in the extract involved in the reduction of silver ions and stabilization process of AgNPs [40,53,54,55,56,57].

**Figure 3:**
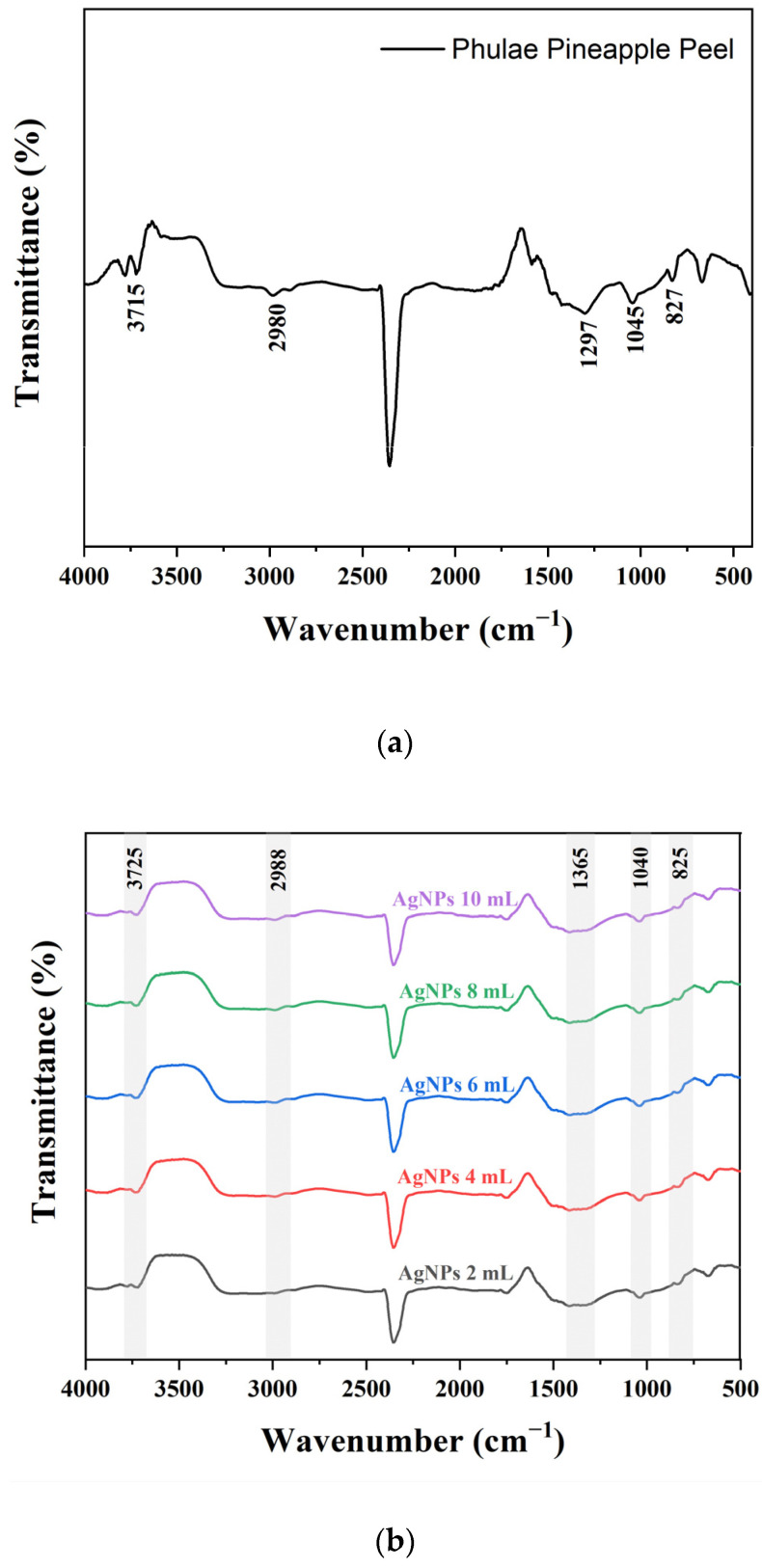

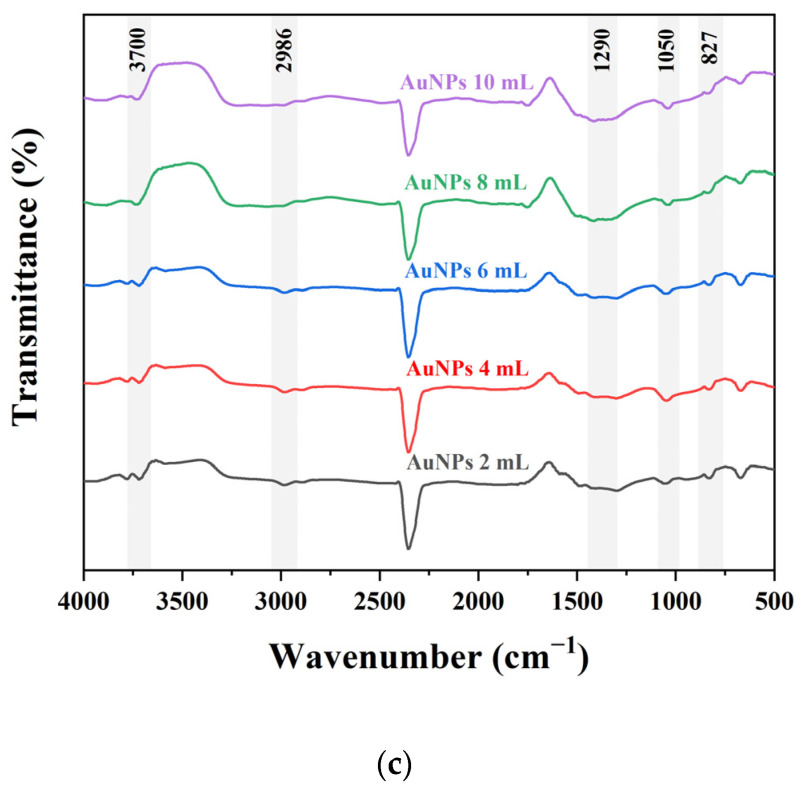
FTIR spectra of (**a**) Phulae pineapple peel, (**b**) AgNPs, and (**c**) AuNPs at different PPA quantities.

FTIR was performed to identify the functional groups on Phulae pineapple peel, which are involved in the reduction of metallic ions and the stabilization of nanoparticles, and the results are shown in Figure 3a–c. The FTIR spectra of Phulae pineapple peel exhibited characteristic peaks at around 3715, 2980, 1297, 1045, and 827 cm^−1^. In the spectra of synthesized nanoparticles, these bands shift to around 3725, 2988, 1365, 1040, and 825 cm^−1^ for AgNPs, and 3700, 2986, 1290, 1050, and 827 cm^−1^ for AuNPs. The peak at 3725–3700 cm^−1^ could be due to the stretching vibration of O-H [58,59]. The peak at 2988–2980 cm^−1^ was assigned to alkane C-H stretching vibrations [60]. The peak at 1365–1290 cm^−1^ was assigned to C-H bending [61]. The peak at 1050–1040 cm^−1^ was assigned to the stretching and deformation of aromatic C-O groups [62]. The peak at 827–825 cm^−1^ was assigned to illustrate the angular deformation of aromatic hydrogen [63]. All the aforementioned vibration bands are caused by the presence of phytochemical substances present in Phulae pineapple peel, which contains functional groups that may have aided the reduction and stability of the nanoparticles.

**Figure 4:**
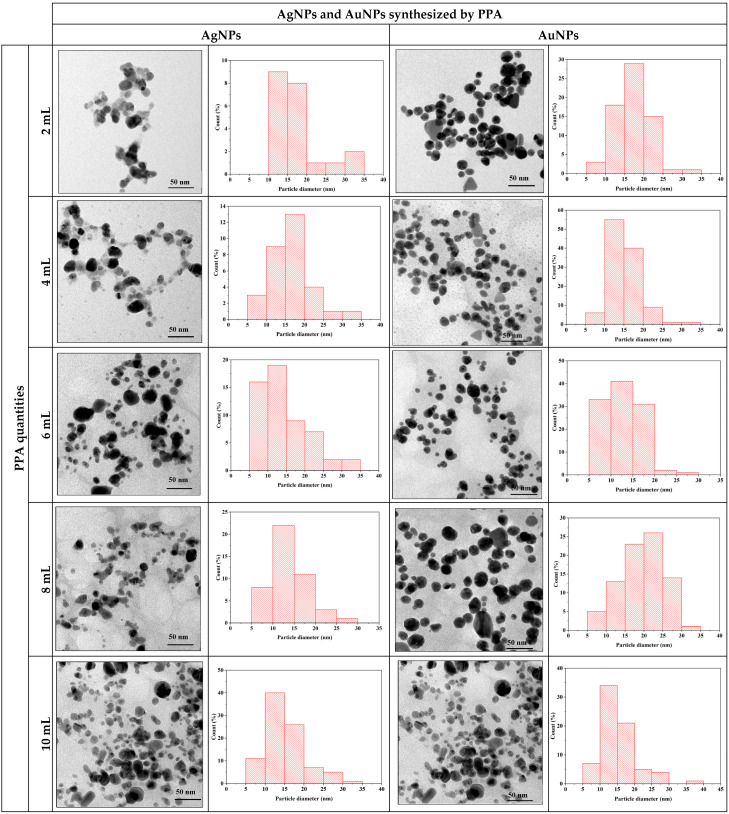
TEM image of biosynthesized AgNPs and AuNPs at different PPA quantities.

TEM images reveal that AgNPs and AuNPs synthesized using PPA predominantly exhibit spherical morphologies, as shown in Figure 4. TEM imaging revealed that the average particle size for AgNPs and AuNPs was between 10 and 30 nm. The average particle sizes of AgNPs and AuNPs synthesized with 10 mL and 6 mL PPA quantities are the smallest overall.

**Figure 5:**
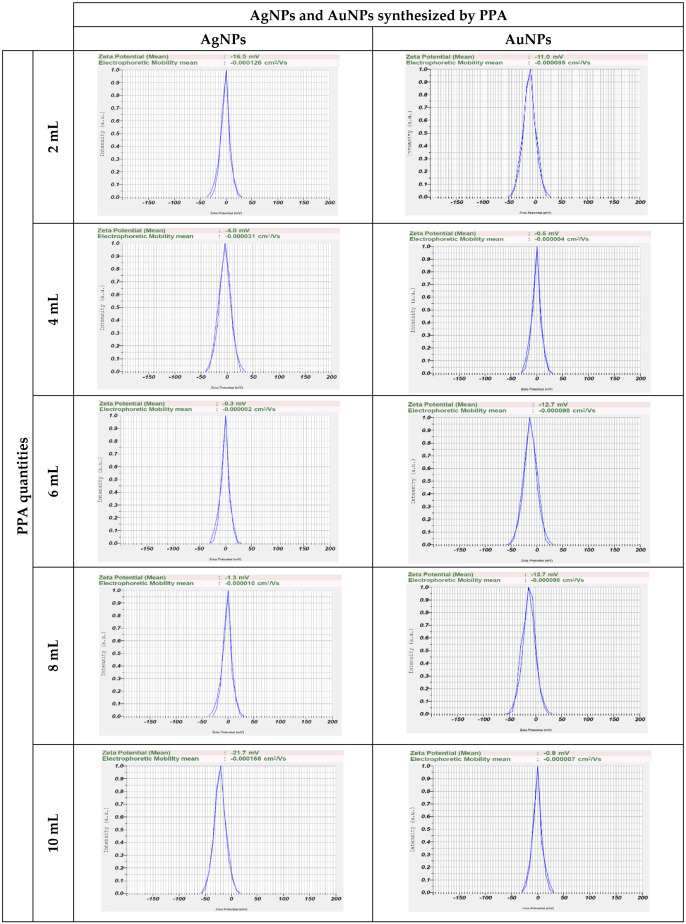
Zeta potential values of biosynthesized AgNPs and AuNPs at different PPA quantities.

Zeta potential is a measure of the effective electric charge on the surface of a nanoparticle; it is often used to analyze the stability of colloidal solutions. The range for stable and unstable suspensions are frequently defined as −30 mV to 30 mV, which means that particles having zeta potentials less than −30 mV or greater than 30 mV are typically considered stable. If an unstable nanoparticle suspension has a zeta potential value greater than −15 mV, it indicates a suspension at the threshold of agglomeration and the formation of larger particles. Coagulation or flocculation is most rapid when the zeta potential is 0 mV ± 3 mV [64,65]. The zeta potential values of AgNPs and AuNPs synthesized using PPA at different PPA quantities (Figure 5) are in the range of −20.7 to −0.3 mV and −12.7 to −0.5 mV, respectively. The colloidal solution of both AgNPs and AuNPs was found to be stable for 1 week, and all particles were not settled down to the bottom (no aggregate). AgNPs precipitated rapidly at 6 and 8 mL PPA quantities, as did AuNPs at 4 and 10 mL PPA quantities. On the contrary, AgNPs at PPA quantities of 10 mL and AuNPs at PPA quantities of 6 mL have the slowest sedimentation without aggregate. It may be inferred that quantities of 10 mL for AgNPs and 6 mL for AuNPs have the greatest colloid solution stability.

### 2.2. Electrochemical Properties of AgNPs and AuNPs Modified Electrodes

**Figure 6:**
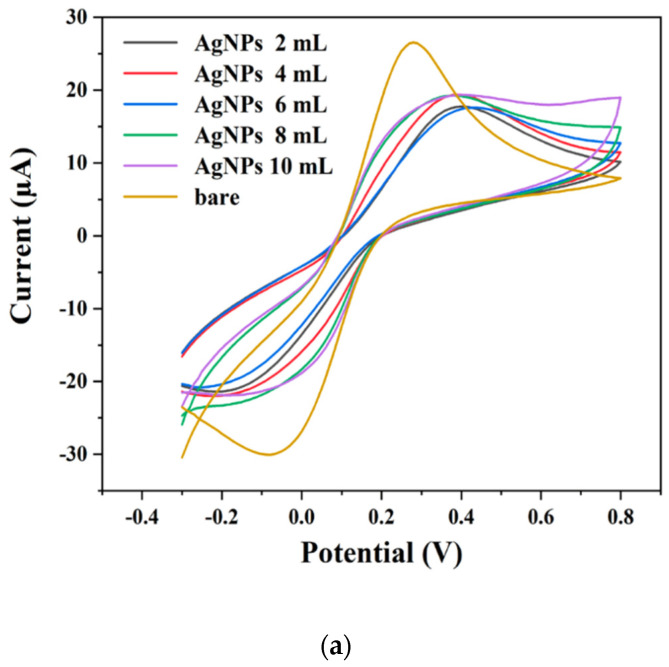

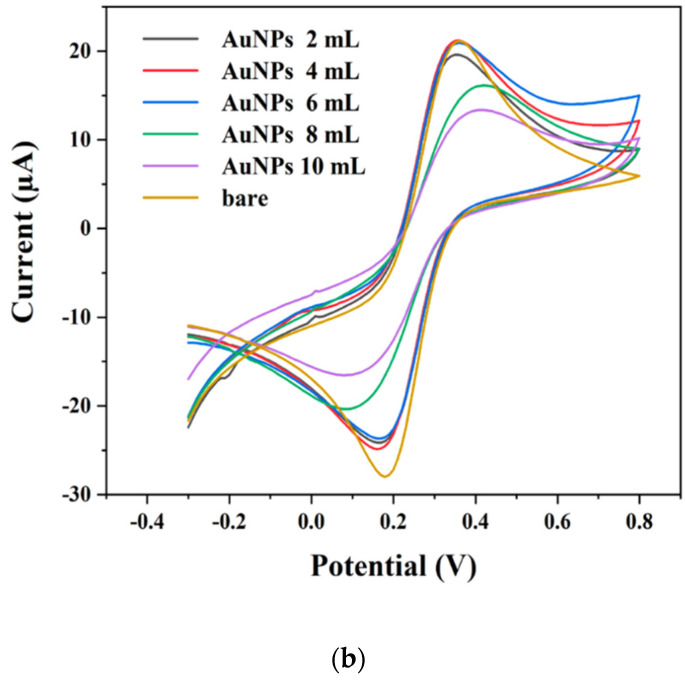
CV curves of bare SPCE with (**a**) AgNPs/SPCE and (**b**) AuNPs/SPCE. The scan rate is 0.1 Vs^−1^ in 5 mM K_3_Fe (CN)_6_ and 5 mM KCl in PBS (pH 7.4).

The electrochemical properties of both bare screen-printed carbon electrodes (SPCE) and modified electrodes (AgNPs/SPCE and AuNPs/SPCE) were evaluated by comparing their electrochemical responses using cyclic voltammetry (CV) in a potassium chloride (KCl) solution with a 5 mM concentration of the redox probe [Fe (CN)_6_]^3−/4−^. The CV curves depicted in Figure 6a,b demonstrate that both the bare SPCEs and the modified electrodes exhibit reversible redox reactions, indicative of effective electron transfer at the electrode surfaces. The peak currents observed for the AgNPs/SPCE electrodes, prepared with 10 mL of phosphoric acid (PPA), and AuNPs/SPCE electrodes, prepared with 6 mL of PPA, were significantly higher than those recorded for the bare SPCE [66]. This enhancement can be attributed to several factors: firstly, the incorporation of nanoparticles increases the effective surface area of the electrodes, allowing for greater accessibility for the redox probe to the electrode surface. Secondly, the nanoparticles serve as catalytic sites that facilitate more efficient electron transfer processes. The morphology of the nanoparticles, as confirmed by transmission electron microscopy (TEM), indicates the presence of small, well-dispersed particles, which enhances the electroactive surface area. Additionally, the stability of the zeta potential values suggests that the modified electrodes maintain a favorable dispersion of nanoparticles, contributing to their electrochemical activity. These findings highlight the potential of using AgNPs and AuNPs to enhance the electrochemical performance of modified electrodes, paving the way for their application in the detection of biomolecules, such as human serum albumin (HSA). Prior to detection, antibodies can be immobilized on the AgNPs/SPCE and AuNPs/SPCE, as outlined in Section 3.7.

### 2.3. Electrochemical Detection of HSA

**Figure 7:**
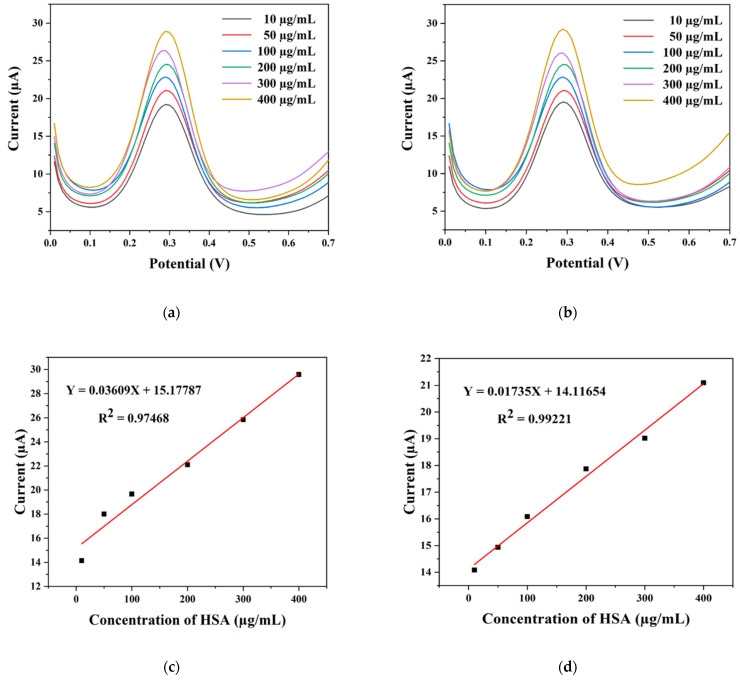
SWV curves of (**a**) AgNP/SPCE/antibody HSA and (**b**) AuNP/SPCE/antibody HSA for detection of different HSA concentrations (10–400 μm/mL). The relationship between the current vs. HSA concentrations for (**c**) AgNP/SPCE/antibody HSA and (**d**) AuNP/SPCE/antibody HSA.

For the detection of human serum albumin (HSA), we used square-wave voltammetry (SWV) measurements, which are more sensitive than CV and are applicable to the qualitative determination of analyte concentrations. The different concentrations of HSA responses on the AgNP/SPCE/antibody HSA and AuNP/SPCE/antibody HSA electrodes are shown in Figure 7a,b, Within the investigated potential range (0–0.7 V), measurements revealed a peak position for the oxidation of HSA at −0.29 V. The graph shows that the concentration of HSA increased, but the position of the oxidation peak did not change, and it was found that current is related to the concentration of solution in detection. Figure 7c,d show a linear relationship between the peak current and HSA concentration obtained (HSA concentration of 10–400 μm/mL), with the linear regression equation being defined by Y = 0.03609X + 15.17787 (R^2^ = 0.97468) for AgNP/SPCE/antibody HSA electrodes and Y = 0.01735X + 14.11654 (R^2^ = 0.99221) for AuNP/SPCE/antibody HSA electrodes. Hence, the relative current measured in SWV demonstrated conclusively that HSA detection was successful.

## 3. Materials and Methods

### 3.1. Materials

Hydrogen tetrachloroaurate (III) hydrate (HAuCl_4_·3H_2_O, 99.9%), anti-human serum albumin antibody, and human serum albumin (HSA) were purchased from Sigma Aldrich. Silver nitrate (AgNO_3_, 99.9%) was purchased from QRëC. Phosphate buffer (0.1 M PBS, pH 7.4) was purchased from Apsalagen Co., Ltd. Screen-printed carbon electrodes (SPCE) and potassium ferricyanide (K_3_[Fe(CN)_6_]) were purchased from Quasense Co., Ltd., Thailand. Phulae pineapple was purchased from Iyara Market in Pathumthani Province, Thailand.

### 3.2. Preparation for Phulae Pineapple Peel Extract (PPA)

To obtain the fruit peel extract, Phulae pineapple peels were washed and cut into smaller pieces, and then were divided into two portions. For the first portion, 20 g of peel was added to 100 mL of deionized water (DI) and boiled. From the moment it started boiling, we waited for about 45–60 min and left it to cool fully covered. After that, the peel was removed by filtration to afford the extract. The peel extract was stored at 4 °C prior to use. For the second portion, it was dried at 80 °C. After drying completely, we crushed the pineapple peel and put it in a humidity chamber to be analyzed using FTIR to verify the presence of any phytochemicals in the plant extract.

### 3.3. Synthesis of Nanoparticles

AgNPs were synthesized by mixing 50 mL of AgNO_3_ solution with a concentration of 3 mM with 2, 4, 6, 8, and 10 mL of PPA. The reaction mixture was magnetically stirred at 60 °C for 1 h, with the formation of AgNPs being observed by a color change to orange-brown. AuNPs were synthesized by mixing 27 mL of HAuCl_4_·3H_2_O solution with a concentration of 1 mM with 2, 4, 6, 8, and 10 mL of PPA. The reaction mixture was magnetically stirred at 100 °C for 15 min, with the formation of AuNPs being observed by a color change to purple. Both samples were taken to undergo UV-vis analysis, and the solution was centrifuged at 10,000 rpm for 15 min to remove any excess reagents and silver ions. The supernatant was discarded, and the resulting pellet was washed with doubly distilled water and then again centrifuged. This process was repeated three times. Subsequently, the purified pellets of synthesized AgNPs and AuNPs were dried at 80 °C in a vacuum oven before analysis by XRD and FTIR.

### 3.4. Characterization of Synthesized Nanoparticles

The optical absorbance nanoscale of the formed AgNPs and AuNPs was confirmed using UV-vis spectrophotometry (Thermo Scientific, GENESYS 10S UV-Vis) spanning a 300–800 nm wavelength range. The X-ray diffraction (XRD) patterns of crystalline structures were measured using a powder X-ray diffractometer (Bruker, D2 PHASER) equipped with CuKα radiation (1.54 Å) in the range of 20–80° in 2θ angles with a scan speed of 2°/min, voltage of 30 kV, and current of 10 mA. To detect organic functional groups contained in powdered Phulae pineapple peel samples and on the surface of nanoparticles, Fourier transform infrared spectrophotometer (FTIR) examinations were performed throughout a 4000–400 cm^−1^ range (Shimadzu, IRSpirit). The samples were prepared as KBr disks. Morphology was investigated using a transmission electron microscope (TEM) (JEOL, JEM-2010). The sample was dispersed in water at a controlled temperature of 25 °C, with the scattering angle set to 90° and the viscosity of the dispersion medium fixed at 0.89 mPa.s (Horiba, Nanopartica sz-100).

### 3.5. Measurements of Electrochemical Responses

Electrochemical experiments were performed using a PalmSens 4 with the PSTrace software 5.9 instrument. A screen-printed carbon electrode (SPCE) consists of three electrodes: the working electrode, a reference electrode (Ag/AgCl), and a counter electrode (carbon bases). Cyclic voltammetry (CV) and square-wave voltammetry (SWV) were used to measure the responses of the modified electrodes.

### 3.6. Preparation of Electrodes Modified with Nanoparticles

The electrode was prepared by dropping 3 μL of nanoparticles onto a screen-printed carbon electrode (SPCE) at the working electrode, which was then dried for 10 min at 40 °C (or at room temperature until it dried) before being used in detection experiments. The electrochemical response of modified electrodes, namely, AgNPs/SPCE and AuNPs/SPCE, was investigated using cyclic voltammetry (CV) with the following parameters: they were measured from 0.4–0.8 V at a scan rate of 0.1 V s^−1^ in 5 mM K_3_Fe (CN)_6_ and 5 mM KCl in PBS (pH 7.4).

### 3.7. Procedure for Detection of HSA

Select the electrode in Section 3.6 that provides the highest electric current signal. Coat the working electrode with 2 μL of the serum albumin antibody and then allow it to dry at room temperature (also called AgNPs/SPCE/antibody (HSA) and AuNPs/SPCE/antibody (HSA)). Next, prepare human albumin solutions at 10, 50, 100, 200, 300, and 400 μg/mL, mix with electrolyte, and drop 5 μL onto AgNPs/SPCE/antibody (HSA) and AuNPs/SPCE/antibody (HSA) electrodes. Subsequently, the response was evaluated using square-wave voltammetry (SWV) at a frequency of 10.0 Hz and an amplitude of 0.05 V, with a measurement range of 0.0 to 0.7 V.

## 4. Conclusions

This study illustrates the feasibility and efficacy of electrochemical methods combined with AgNP- and AuNP-modified electrodes for sensitivity, high stability, good analytical performance, and specific HSA detection. This will lead to the development of efficient and inexpensive diagnostic tools for various kinds of diseases and health conditions.

## Data Availability

The datasets generated and/or analyzed during the current study are not publicly available but are available from the corresponding author upon reasonable request.
